# Platelet rich fibrin augmented tympanoplasty versus cartilage tympanoplasty: a randomized clinical trial

**DOI:** 10.1007/s00405-024-08819-2

**Published:** 2024-07-23

**Authors:** Ahmad Muhammad Al-Arman, Waleed Moneir, Hazem Emam Amer, Hisham Atef Ebada

**Affiliations:** Department of Otorhinolaryngology, Mansoura University, Mansoura, 35511 Egypt

**Keywords:** Tympanoplasty, Chronic otitis media, Fascia, Cartilage, Platelet rich fibrin

## Abstract

**Objectives:**

The aim of the current study was to evaluate the efficacy of PRF-augmented fascia tympanoplasty versus cartilage tympanoplasty in repair of large TM perforations.

**Methods:**

This randomized clinical trial included 156 patients with dry large tympanic membrane perforations. Patients were randomly allocated into 2 groups, cartilage tympanoplasty group (*n* = 77) and platelet rich fibrin (PRF) augmented tympanoplasty group (*n* = 79). Graft take rates, hearing outcomes, operative time, and postoperative complications were documented and compared.

**Results:**

Graft take rate was 96.1% in the cartilage group and 93.7% PRF group with no statistically significant difference. Operative time was significantly longer in the cartilage group. No differences in the hearing outcomes and postoperative complications were reported.

**Conclusion:**

Application of PRF on the fascia in tympanoplasty promotes healing of the tympanic membrane. PRF is safe, cheap, readily available, and easily prepared and applied. It increases the success rates of large tympanic membrane perforations without the need for cartilage grafts.

## Introduction

Tympanoplasty is a common procedure performed to obtain an intact tympanic membrane (TM) and to improve hearing [1]. Large, subtotal, and total TM perforations are the most challenging due to access difficulty, and lack of adequate support to the graft [2]. These perforations are more likely to fail, Therefore, there is an ongoing research to find the optimum technique to improve outcomes [3, 4].

Several materials are used for TM reconstruction including temporalis fascia, tragal or conchal cartilage, perichondrium, periosteum, skin, and fat [5]. Temporalis fascia is the most widely applied due to its availability at the same surgical field, comparable thickness to the TM, low metabolic rates and good postoperative compliance [1].

Application of cartilage in tympanoplasty is gaining popularity amongst otologists. Its rigidity has obvious benefits in preventing TM retraction [6]. It was reported that success rates for cartilage are higher than facia especially in large perforations [7, 8].

Healing of the TM after tympanoplasty is a complex process that involves epithelial proliferation and migration, fibroblasts proliferation, angiogenesis, and tissue remodeling [9]. Recent studies in molecular biology and tissue engineering have highlighted the significance of growth factors in the process of wound healing. These natural bio-mediators play a crucial role in regulating various cellular activities essential for healing, including cell proliferation, differentiation, and chemotaxis [10–12].

Platelet-rich fibrin (PRF) was introduced by Choukroun et al. [13] in 2001. PRF comprises a group of cytokines, glycanic chains, and structural glycoproteins embedded in a fibrin framework. These substances have a pivotal role in tissue healing [11]. Platelets produce cytokines and growth factors such as tumor growth factor β, platelet-derived growth factor, insulin-like growth factor-1, and vascular endothelial growth factor [14].

The aim of the current study was to evaluate the efficacy of PRF-augmented fascia tympanoplasty versus cartilage tympanoplasty in repair of large TM perforations.

## Patients and methods

This parallel double-blinded randomized clinical trial was performed in the Otorhinolaryngology Department, Mansoura University Hospital, Egypt, over a period of 4 years (June 2019 – June 2023). Informed written consents were obtained from all participants, and the study was approved by the Mansoura Faculty of Medicine Institutional Review Board (MFM-IRB: MS.19.04.576). This randomized clinical trial was registered at ClinicalTrials.gov (NCT05967845).

### Patients

This study included 156 adult patients (≥ 18 years) with dry large tympanic membrane perforations. Patients were randomly allocated into 2 groups, cartilage tympanoplasty group (*n* = 77) and platelet rich fibrin (PRF) augmented tympanoplasty group (*n* = 79), using the block randomization method. Exclusion criteria included recurrent perforations after previous tympanoplasty, active middle ear inflammation, cholesteatoma, and ossicular disruption or fixation diagnosed intra-operatively.

### Preoperative assessment

The TM was examined by using a mm Karl Storz rigid endoscope with 0 angle of view to provide an excellent visualization. The size of the perforation was assessed according to Saliba [15] classification (Table 1). Additionally, the middle ear mucosa was inspected to exclude active infection.

**Table 1:** Saliba classification of tympanic membrane perforation size

Perforation	Size in percent	Tympanic membrane quadrant affected
Small	Less than 25%	Less than one quadrant size
Medium	More than 25% - less than 50%	More than one quadrant size – less than 2 quadrant size
Large	More than 50% - not total	More than 2 quadrant size – not total
Total	100% - or total	Completely 4 quadrant size

Preoperative pure-tone audiograms were obtained. Air conduction and bone conduction thresholds were tested at 0.5, 1, 2, 4 kHz by supra- aural headphone for air conduction and by bone vibrator for bone conduction.

### Surgical techniques

Patients were positioned and draped in the standard manner for tympanoplasty operations. The post-auricular approach was performed in all participants. In both groups a piece of temporalis fascia (1 × 2 cm.) was harvested. In the cartilage tympanoplasty group, a piece of cartilage was harvested from the concha. Fascia and cartilage grafts were harvested through the same post-auricular incision.

### Preparation of the PRF clot

It was prepared in the operative theater just after induction of anesthesia. Ten milliliters of the patient’s own blood were withdrawn into a capped tube without application of anticoagulant.

**Fig. 1:**
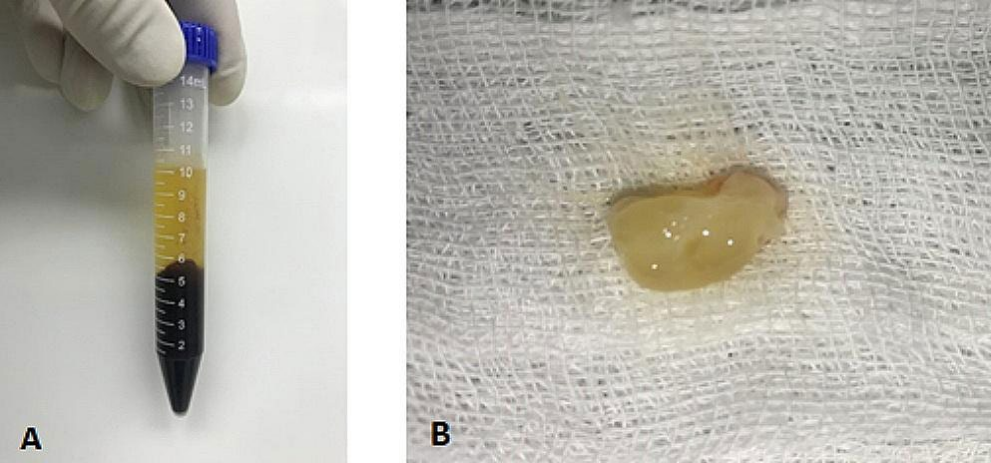
PRF preparation; **A**: Blood sample after centrifugation. **B**: PRF clot on a sterile gauze

The tube was then centrifuged using a centrifuge machine for 10 min at 3000 rpm. The resultant product consists of three layers; the top layer is composed of acellular platelet poor plasma, the middle layer is the PRF clot, and the bottom layer is composed of red blood cells. The PRF clot was separated and put on a sterile gauze to be ready for placement (Fig. 1).

### Placement of the grafts

**Fig. 2:**
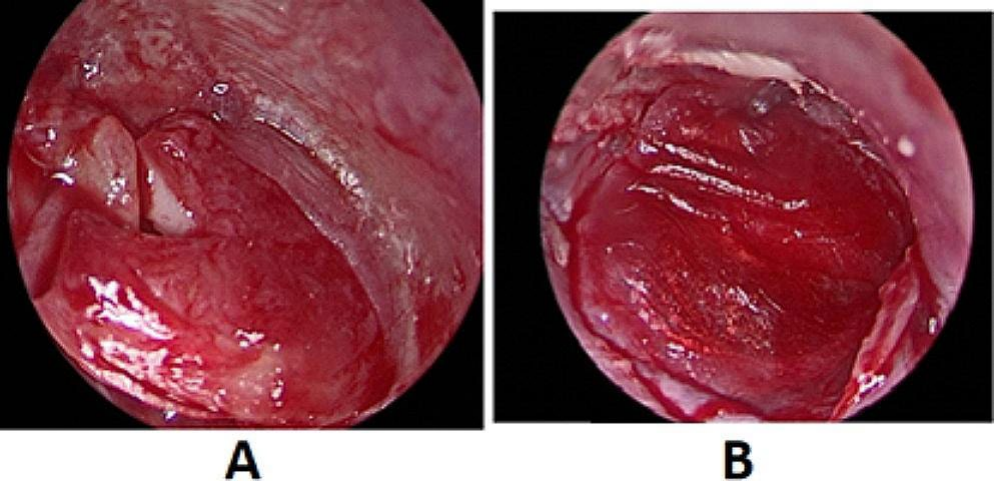
**A**: Placement of cartilage shield graft. **B**: Placement of temporalis fascia graft over the cartilage graft

In the cartilage tympanoplasty group, the cartilage graft was reshaped to fit in the middle ear. A small triangular piece was removed superiorly to look like a shield to fit the malleus. The cartilage graft was put medial to malleus then temporalis fascia graft is put by over-underlay technique, under the annulus rim and over the malleus (Fig. 2).

**Fig. 3:**
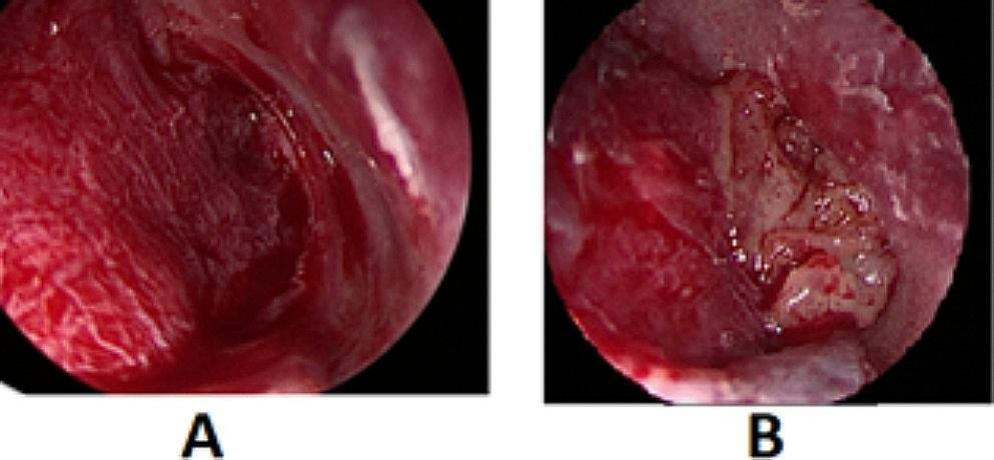
**A**: Placement of temporalis fascia graft. **B**: Application of the PRF clot

In the PRF-augmented tympanoplasty group, the temporalis fascia graft was placed in the middle ear over the malleus handle and under the annulus and TM remnant. Once the graft was in good position, the tympano-meatal flap was returned to its anatomic position (Fig. 3-A).

The PRF clot was applied to the fitted graft (Fig. 3-B). Pieces of gelfoam were put over the drum remnant and graft-PRF complex to hold it in position.

### Study outcomes

**Fig. 4:**
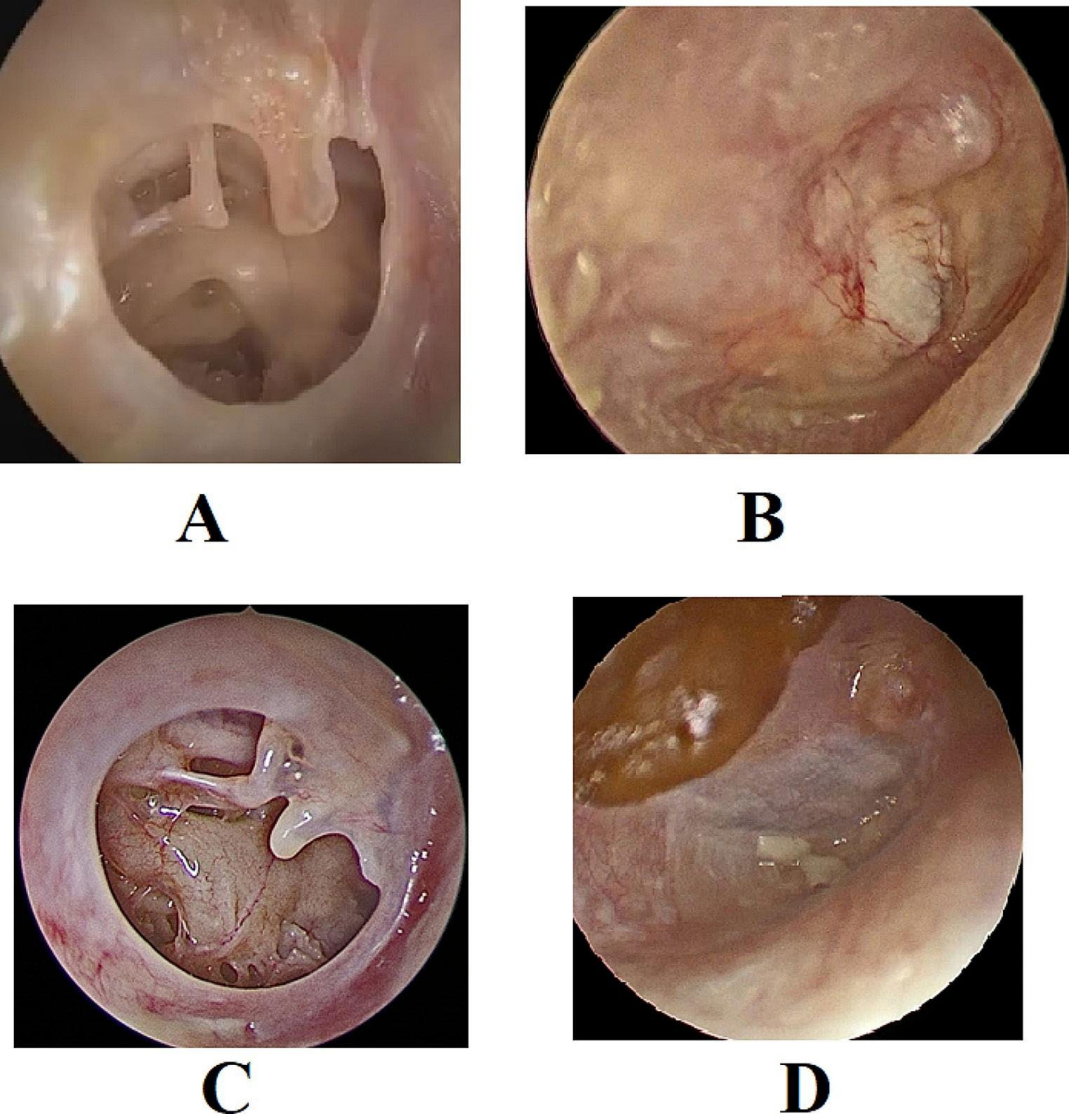
Successful outcome of cartilage tympanoplasty; **A**: preoperative photo, **B**: postoperative photo (3 months after surgery). Successful outcome of PRF augmented fascia tympanoplasty; **C**: preoperative photo, **D**: postoperative photo (3 months after surgery)

The primary outcome was graft take rate. This was assessed 3 months after surgery and was defined as an intact tympanic membrane with no residual perforation (Fig. 4).

Secondary outcomes included operative time, hearing outcomes, and postoperative complications. Pure tone audiometry was performed 3 months after surgery and was compared to the preoperative values. Post-operative complications were documented.

## Results

The present study involved 156 patients with a dry large TM perforation. Seventy-seven patients underwent cartilage tympanoplasty, and 79 underwent PRF augmented tympanoplasty. Table (2) shows the demographic data of the patients. No statistically significant differences were found between groups regarding age and gender.

**Table 2:** Demographic data among cartilage and PRF groups

Sociodemographic data	Cartilage group (*n* = 77)	PRF group (*n* = 79)	Test of significance (*p* value)
**Age (years)**	33.61 ± 9.03	30.75 ± 8.60	t = 1.29
Mean ± SD	19–50	18–50	*P* = 0.203
**Gender**			
Male	35 (45.5%)	43 (54.4%)	χ^2^ = 0.40
Female	42 (54.5%)	36 (45.6%)	*p* = 0.527
**Size of perforation according to Saliba classification**	Large perforation: 77 (100%)	Large perforation: 79 (100%)	

t: student t- test, χ^2^ : Chi square test

There was no statistically significant difference between the two groups regarding the preoperative hearing levels. Table (3) shows the air conduction (AC) thresholds, bone conduction (BC) thresholds, and air bone gap (ABG). Similarly, regarding the postoperative audiometric data, no difference was reported.

**Table 3:** Comparison between both groups regarding preoperative and postoperative audiometric data (using the paired t test)

			Cartilage group (*n* = 77)	PRF group (*n* = 79)	*p* value
**Preoperative audiometry**	**AC**	**0.5 K**	43.87 ± 8.80	45.19 ± 9.71	*P* = 0.575
		**1 K**	42.35 ± 9.00	42.22 ± 8.42	*P* = 0.951
		**2 K**	38.96 ± 8.15	39.25 ± 7.70	*P* = 0.888
		**4 K**	43.29 ± 11.27	39.94 ± 9.89	*P* = 0.214
		**Average** **AC**	42.12 ± 6.45	41.65 ± 6.84	*P* = 0.779
	**BC**	**0.5 K**	16.90 ± 5.62	14.56 ± 6.04	*P* = 0.117
		**1 K**	15.23 ± 6.17	14.28 ± 5.60	*P* = 0.527
		**2 K**	16.06 ± 5.01	14.28 ± 5.28	*P* = 0.175
		**4 K**	17.90 ± 7.35	15.18 ± 5.97	*P* = 0.112
		**Average BC**	16.52 ± 4.71	14.58 ± 4.11	*P* = 0.086
	**ABG**	**0.5k**	26.96 ± 5.78	30.62 ± 8.03	*P* = 0.053
		**1k**	27.12 ± 7.57	27.94 ± 7.27	*P* = 0.667
		**2k**	22.90 ± 6.43	24.97 ± 6.76	*P* = 0.219
		**4k**	25.38 ± 7.81	24.75 ± 7.56	*P* = 0.743
		**Average ABG**	25.59 ± 4.84	27.07 ± 5.53	*P* = 0.266
**Postoperative audiometry**	**AC**	**0.5 K**	24.00 ± 6.66	24.00 ± 8.09	*P* = 1.0
		**1 K**	24.06 ± 6.71	21.59 ± 7.06	*P* = 0.160
		**2 K**	25.19 ± 7.14	21.78 ± 6.73	*P* = 0.056
		**4 K**	25.93 ± 7.60	23.12 ± 6.81	*P* = 0.127
		**Average AC**	24.79 ± 6.03	22.62 ± 6.06	*P* = 0.159
	**BC**	**0.5 K**	15.96 ± 4.92	15.37 ± 5.85	*P* = 0.666
		**1 K**	15.93 ± 5.39	13.56 ± 4.55	*P* = 0.064
		**2 K**	16.87 ± 4.70	14.09 ± 5.34	*P* = 0.033
		**4 K**	17.52 ± 5.03	15.40 ± 5.36	*P* = 0.113
		**Average BC**	16.57 ± 4.23	14.61 ± 3.96	*P* = 0.062
	**ABG**	**0.5k**	8.03 ± 4.29	8.62 ± 6.62	*P* = 0.676
		**1k**	8.13 ± 5.20	8.03 ± 5.61	*P* = 0.943
		**2k**	8.32 ± 6.34	7.68 ± 5.72	*P* = 0.678
		**4k**	8.42 ± 5.93	7.72 ± 5.58	*P* = 0.631
		**Average ABG**	8.22 ± 4.97	8.01 ± 5.65	*P* = 0.876

AC: air conduction. BC: Bone conduction. ABG: Air bone gap

Regarding hearing outcomes, there was significant improvement in hearing after surgery in both groups. Table (4) shows a comparison between preoperative and postoperative audiometric data in both groups. A statistically significant improvement in AC thresholds as well as air bone gaps was reported in both groups.

**Table 1:** Comparison between pre-operative and post-operative audiometric data in both groups (using the paired t test)

		Cartilage group (*n* = 77)		Paired t test (*p* value)	PRF group (*n* = 79)		*p* value
		Pre	Post		Pre	Post	
**AC**	**0.5 K**	43.87 ± 8.80	24.00 ± 6.66	t = 17.07 *P* ≤ 0.001*	45.19 ± 9.71	24.00 ± 8.09	*P* ≤ 0.001*
	**1 K**	42.35 ± 9.00	24.06 ± 6.71	t = 11.61 *P* ≤ 0.001*	42.22 ± 8.42	21.59 ± 7.06	*P* ≤ 0.001*
	**2 K**	38.96 ± 8.15	25.19 ± 7.14	t = 9.53 *P* ≤ 0.001*	39.25 ± 7.70	21.78 ± 6.73	*P* ≤ 0.001*
	**4 K**	43.29 ± 11.27	25.93 ± 7.60	t = 12.42 *P* ≤ 0.001*	39.94 ± 9.89	23.12 ± 6.81	*P* ≤ 0.001*
	**Average AC**	42.12 ± 6.45	24.79 ± 6.03	t = 19.62 *P* ≤ 0.001*	41.65 ± 6.84	22.62 ± 6.06	*P* ≤ 0.001*
**BC**	**0.5 K**	16.90 ± 5.62	15.96 ± 4.92	t = 1.07 *P* = 0.294	14.56 ± 6.04	15.37 ± 5.85	*P* = 0.374
	**1 K**	15.23 ± 6.17	15.93 ± 5.39	t = 0.891 *P* = 0.380	14.28 ± 5.60	13.56 ± 4.55	*P* = 0.431
	**2 K**	16.06 ± 5.01	16.87 ± 4.70	t = 0.989 *P* = 0.331	14.28 ± 5.28	14.09 ± 5.34	*P* = 0.690
	**4 K**	17.90 ± 7.35	17.52 ± 5.03	t = 0.566 *P* = 0.576	15.18 ± 5.97	15.40 ± 5.36	*P* = 0.809
	**Average BC**	16.52 ± 4.71	16.57 ± 4.23	t = 0.142 *P* = 0.888	14.58 ± 4.11	14.61 ± 3.96	*P* = 0.937
**ABG**	**0.5k**	26.96 ± 5.78	8.03 ± 4.29	t = 23.42 *P* ≤ 0.001*	30.62 ± 8.03	8.62 ± 6.62	*P* ≤ 0.001*
	**1k**	27.12 ± 7.57	8.13 ± 5.20	t = 14.88 *P* ≤ 0.001*	27.94 ± 7.27	8.03 ± 5.61	*P* ≤ 0.001*
	**2k**	22.90 ± 6.43	8.32 ± 6.34	t = 12.59 *P* ≤ 0.001*	24.97 ± 6.76	7.68 ± 5.72	*P* ≤ 0.001*
	**4k**	25.38 ± 7.81	8.42 ± 5.93	t = 14.28 *P* ≤ 0.001*	24.75 ± 7.56	7.72 ± 5.58	*P* ≤ 0.001*
	**Average ABG**	25.59 ± 4.84	8.22 ± 4.97	t = 21.43 *P* ≤ 0.001*	27.07 ± 5.53	8.01 ± 5.65	*P* ≤ 0.001*

AC: air conduction. BC: Bone conduction. ABG: Air bone gap. *significant *p* ≤ 0.05

Regarding the graft take rate, it was slightly higher in the cartilage group (96.1%) than in PRF group (93.7%) but with no statistically significant difference (*P* = 0.831) (Table 5).

The mean operative time for cartilage group (80.81 ± 7.86 min) was significantly longer than that of PRF group (72.97 ± 6.45), (*P* ≤ 0.001).

**Table 5:** Study outcomes: graft take rate, operative time and infection rates

Study outcomes		Cartilage group (*n* = 77)	PRF group (*n* = 79)	Test of significance (*p* value)
**Graft take rate**	Healed No (%)	74 (96.1%)	74 (93.7%)	Chi-square = 3.243 *P* = 0.831
	Not healed No (%)	3 (3.9%)	5 (6.3%)	
**Operative time** Mean ± SD		80.81 ± 7.86	72.97 ± 6.45	t = 4.33 *P* ≤ 0.001*
**Infection**	Yes	5 (6.5%)	2 (2.5%)	FET *p* = 0.789
	No	72 (93.5%)	77 (97.5%)	
**Duration of infection** Mean ± SD		7.23 ± 1.71	7.31 ± 1.74	t = 0.199 *P* = 0.843

FET: Fischer exact test, *significant *p* ≤ 0.05

Regarding postoperative complications, postoperative infection was reported more frequently in the cartilage group (*n* = 5) than in the PRF group (*n* = 2), however, this was statistically insignificant (*p* = 0.789). Infection was successfully managed in all patients with appropriate antibiotic ear drops. No other intraoperative, or postoperative complications in the form of canal stenosis, lateralization, anterior blunting, or ear protrusion, were reported in both groups.

## Discussion

To overcome surgical challenges associated with large tympanic membrane perforations, several techniques and approaches have been adopted with variable outcomes. These techniques include three-point fix technique [16], loop overlay tympanoplasty with a superiorly based tympano-meatal flap [17], sandwich graft tympanoplasty [18] and double flap technique [19].

More frequently, cartilage grafts are commonly used to improve success rates. Iacovou et al. [20] performed a large systemic review on 1,286 patients and reported significant higher success rates in cartilage group (92.4%) in comparison to temporalis fascia group (84.3%). Similarly, Duckert et al. [21] reported the use of cartilage in tympanoplasty in 294 patients with 96.9% success rate. In the current work, the success rate in the cartilage group was 96.1%.

Despite higher success rates, cartilage tympanoplasty has some drawbacks. One possible drawback is that the opacity of the graft could make it difficult to detect any recurrent or residual cholesteatoma, as well as middle ear effusion. [22]. Concerns that the stiffness and mass of cartilage grafts may adversely affect hearing outcome of tympanoplasty [23]. In addition, cartilage harvesting and placement increase the operative time [22].

Our results showed that application of the PRF clot on the fascia graft enhances the healing process. Consequently, high success rates, similar to cartilage tympanoplasty, are achieved without the drawbacks of cartilage grafts.

PRF is an autologous product rich in platelet concentrate (> 1,000,000 platelets/ml), along with white blood cells, stem cells, and fibrin matrix [24]. Platelets produce multiple cytokines and growth factors [14]. Growth factors regulate cell proliferation, differentiation, and chemotaxis during the healing process [10, 12]. Moreover, platelets have molecules such as fibrin, fibronectin, and vitronectin, which act as cell adhesion molecules [11, 12].

PRF is applied in multiple medical disciplines including grafts and wounds treatment [11, 25], cardiothoracic, general, plastic, vascular, maxillofacial and dental surgeries [26]. Furthermore, it is also used in otorhinolaryngological procedures such as facial nerve injuries [27], septoplasty [28], turbinate surgery [29], reconstruction of cerebrospinal fluid leaks [30], and pharyngeal closure after total laryngectomy [31].

Additionally, the positive impact of PRF on healing and success rates of tympanoplasty was reported in multiple previous studies [32–35]. Sankaranarayanan et al. [35] reported success in 24 out of 25 patients with application of PRF with fascia compared to only 20 out of 25 patients with fascia alone. Additionally, El-Anwar et al. [36] achieved 84% success rate (21 out of 25 patients) in small dry perforations by application of a PRF hourglass graft as an office procedure. Similarly, Nair et al. [37] reported success rate of 91.6% with application of PRF with temporalis fascia compared to success rate of 66.6% with application of fascia alone. In the current series, the use of PRF with temporalis fascia in tympanoplasty resulted in high success rates (93.7%) with no statistically significant difference between this technique and the cartilage tympanoplasty (96.1%).

Insertion of the temporalis fascia graft in the current work was done by the over-underlay technique. This technique takes the advantages of overlay technique in providing good support and stability to the graft and avoiding medialization [38, 39]. Additionally, the drawbacks of the overlay technique such as anterior blunting, lateralization of the graft and iatrogenic cholesteatoma [40–42] are minimized. Moreover, the over-underlay technique avoids the drawbacks of underlay technique such as decreased mesotympanic space and lack of proper graft support [43, 44].

The operative time in the current work was significantly shorter in the PRF group than in cartilage group. Iacovou et al. [22] reported that in cartilage tympanoplasty surgery, additional 10–15 min are required to obtain the graft.

Interestingly, the rate of postoperative infection in the PRF group was less than that of the cartilage group. Although statistically insignificant, a possible explanation is the presence of white blood cells in high concentrations PRF which may have had a role against infection with its bactericidal effect [45].

PRF-augmented fascia tympanoplasty is easy to perform and has high success rates. PRF is cost-effective and made from the patients’ own blood without risks of adverse effects or aphylactic reaction. Unlike other flaps and techniques, it is not time consuming and with no risk of donor site morbidities [30].

## Conclusion

Application of PRF on the fascia in tympanoplasty promotes healing of the tympanic membrane. PRF is safe, cheap, readily available, and easily prepared and applied. It increases the success rates of large tympanic membrane perforations without the need for cartilage grafts.

## References

[1] Venkatesan D, Umamaheswaran P, Vellikkannu R, Kannan S, Sivaraman A, Ramamurthy S (2021) A comparative study of Temporalis Fascia Graft and full thickness Tragal Island Cartilage Graft in Type 1 Tympanoplasty. Indian J Otolaryngol Head Neck Surg :1–5 10.1007/s12070-021-02459-2 PMC9411460 36032890

[2] Elhendi W (2002) Prognostic factors influencing anatomic and functional outcome in myringoplasty. Acta Otorrinolaringologica Esp 53(10):729–735 10.1016/s0001-6519(02)78369-6 12658839

[3] Scally CM, Allen L, Kerr AG (1996) The anterior hitch method of tympanic membrane repair. Ear nose Throat J 75(4):244–247 8935648

[4] Carr S, Strachan D, Raine C (2015) Factors affecting myringoplasty success. J Laryngol Otol 129(1):23–26 25656157 10.1017/S0022215114003156

[5] Jain A, Samdani S, Sharma MP, Meena V (2018) Island cartilage vs temporalis fascia in type 1 tympanoplasty: a prospective study. Acta Otorrinolaringologica (English Edition) 69(6):311–317 29576202 10.1016/j.otorri.2017.10.004

[6] Mohamad SH, Khan I, Hussain SM (2012) Is cartilage tympanoplasty more effective than fascia tympanoplasty? A systematic review. Otology Neurotology 33(5):699–705 22643445 10.1097/MAO.0b013e318254fbc2

[7] Kazikdas KC, Onal K, Boyraz I, Karabulut E (2007) Palisade cartilage tympanoplasty for management of subtotal perforations: a comparison with the temporalis fascia technique. Eur Arch Otorhinolaryngol 264(9):985–989 17401572 10.1007/s00405-007-0291-3

[8] Ozbek C, Çiftçi O, Tuna EEU, Yazkan Ö, Ozdem C (2008) A comparison of cartilage palisades and fascia in type 1 tympanoplasty in children: anatomic and functional results. Otology Neurotology 29(5):679–683 18580702 10.1097/MAO.0b013e31817dad57

[9] Wang AY, Shen Y, Wang JT, Eikelboom RH, Dilley RJ (2014) Animal models of chronic tympanic membrane perforation: in response to plasminogen initiates and potentiates the healing of acute and chronic tympanic membrane perforations in mice. Clin Translational Med 3:1–3 10.1186/2001-1326-3-5 PMC3987050 24669846

[10] Lou Z, Wang Y (2015) Evaluation of the optimum time for direct application of fibroblast growth factor to human traumatic tympanic membrane perforations. Growth Factors 33(2):65–70 25373361 10.3109/08977194.2014.980905

[11] Dohan DM, Choukroun J, Diss A, Dohan SL, Dohan AJ, Mouhyi J, Gogly B (2006) Platelet-rich fibrin (PRF): a second-generation platelet concentrate. Part II: platelet-related biologic features. Oral Surgery, Oral Medicine, Oral Pathology, Oral Radiology, and Endodontology 101 (3):e45-e50 10.1016/j.tripleo.2005.07.009 16504850

[12] Ehrenfest DMD, Rasmusson L, Albrektsson T (2009) Classification of platelet concentrates: from pure platelet-rich plasma (P-PRP) to leucocyte-and platelet-rich fibrin (L-PRF). Trends Biotechnol 27(3):158–167 19187989 10.1016/j.tibtech.2008.11.009

[13] Choukroun J, Adda F, Schoeffler C, Vervelle A (2001) Une opportunité en paro-implantologie: le PRF. Implantodontie 42:55–62

[14] Zhao Q, Ding Y, Si T (2013) Platelet-rich fibrin in plastic surgery. OA Evidence-Based Med 1(1):3–8

[15] Saliba I (2008) Hyaluronic acid fat graft myringoplasty: how we do it. Clin Otolaryngol Allied Sci 33(6):610–614 10.1111/j.1749-4486.2008.01823.x PMC2691897 19126140

[16] Shim DB, Kim HJ, Kim MJ, Moon IS (2015) Three-point fix tympanoplasty. Acta Otolaryngol 135(5):429–434 25739416 10.3109/00016489.2014.985800

[17] Lee H-Y, Auo H-J, Kang J-M (2010) Loop overlay tympanoplasty for anterior or subtotal perforations. Auris Nasus Larynx 37(2):162–166 19695802 10.1016/j.anl.2009.06.002

[18] Farrior JB (1995) Sandwich graft tympanoplasty: a technique for managing difficult tympanic membrane perforation. Operative Techniques Otolaryngology-Head Neck Surg 6(1):27–32

[19] El-Kholy NA, Salem MA, Rakha AM (2021) Endoscopic single versus double flap tympanoplasty: a randomized clinical trial. Eur Arch Otorhinolaryngol 278(5):1395–1401 32691232 10.1007/s00405-020-06212-3

[20] Iacovou E, Vlastarakos PV, Papacharalampous G, Kyrodimos E, Nikolopoulos TP (2013) Is cartilage better than temporalis muscle fascia in type I tympanoplasty? Implications for current surgical practice. Eur Arch Otorhinolaryngol 270(11):2803–2813 23321796 10.1007/s00405-012-2329-4

[21] Duckert LG, Müller J, Makielski KH, Helms J (1995) Composite autograft shield reconstruction of remnant tympanic membranes. Am J Otology 16(1):21–26 8579173

[22] Iacovou E, Kyrodimos E, Sismanis A (2014) Cartilage shield tympanoplasty: an effective and practical technique. Eur Arch Otorhinolaryngol 271:1903–1908 23999593 10.1007/s00405-013-2679-6

[23] Yung M (2008) Cartilage tympanoplasty: literature review. J Laryngol Otol 122(7):663–672 18312709 10.1017/S0022215108001813

[24] Ehrenfest DD, Sammartino G, Shibli JA, Wang H-L, Zou D-R, Bernard J-P (2013) Guidelines for the publication of articles related to platelet concentrates (platelet-Rich Plasma-PRP, or platelet-rich Fibrin-PRF): the international classification of the POSEIDO. Poseido J 1:17–28

[25] Cortese A, Pantaleo G, Borri A, Caggiano M, Amato M (2016) Platelet-rich fibrin (PRF) in implant dentistry in combination with new bone regenerative technique in elderly patients. Int J Surg case Rep 28:52–56 27689517 10.1016/j.ijscr.2016.09.022 PMC5043401

[26] Eshghpour M, Majidi MR, Nejat AH (2012) Platelet-rich fibrin: an autologous fibrin matrix in surgical procedures: a case report and review of literature. Iran J Otorhinolaryngol 24(69):197–202 24303410 PMC3846193

[27] Cho HH, Lee SC, Jang SJ, Kim SH, Jeong HS, Park JS, Han JY, Lee KH, Cho YB (2009) Effect of platelet Rich plasma on facial nerve regeneration in Acute nerve Injury Model. Korean J Otorhinolaryngology-Head Neck Surg 52(6):486–491

[28] Tutar B, Ekincioglu E, Karaketir S, Berkiten G, Saltürk Z, Arkan ME, Göker AE, Uyar Y (2020) The impact of platelet-rich fibrin (PRF) on olfactory function and pain after septoplasty operations. European Archives of Oto-Rhino-Laryngology:1–6 10.1007/s00405-020-05839-6 32048028

[29] Vieira F, Pierre C, Castro C, Haverroth R (2018) Platelet Rich Fibrin (PRF): an autologous Biomaterial for Turbinectomy Healing assistance. Am J Otolaryngol Head Neck Surg 1(6):1027:27–34

[30] Khafagy YW, Abd Elfattah AM, Moneir W, Salem EH (2018) Leukocyte-and platelet-rich fibrin: a new graft material in endoscopic repair of spontaneous CSF leaks. Eur Arch Otorhinolaryngol 275(9):2245–2252 29982939 10.1007/s00405-018-5048-7

[31] Eid AM, Ebada HA, El-Fattah AMA, Tawfik A (2021) Platelet-rich fibrin: an autologous biomaterial for healing assistance of pharyngeal repair in total laryngectomy. Eur Arch Otorhinolaryngol 278(2):463–470 33009930 10.1007/s00405-020-06404-x

[32] Gür ÖE, Ensari N, Öztürk MT, Boztepe OF, Gün T, Selçuk ÖT, Renda L (2016) Use of a platelet-rich fibrin membrane to repair traumatic tympanic membrane perforations: a comparative study. Acta Otolaryngol 136(10):1017–1023 27192505 10.1080/00016489.2016.1183042

[33] Borie E, Oliví DG, Orsi IA, Garlet K, Weber B, Beltrán V, Fuentes R (2015) Platelet-rich fibrin application in dentistry: a literature review. Int J Clin Exp Med 8(5):7922–7929 26221349 PMC4509294

[34] Cheng G, Ma X, Li J, Cheng Y, Cao Y, Wang Z, Shi X, Du Y, Deng H, Li Z (2018) Incorporating platelet-rich plasma into coaxial electrospun nanofibers for bone tissue engineering. Int J Pharm 547(1–2):656–666 29886100 10.1016/j.ijpharm.2018.06.020

[35] Sankaranarayanan G, Prithviraj V, Kumar V (2013) A study on efficacy of autologous platelet rich plasma in myringoplasty. Online J Otolaryngol 3(3):36–51

[36] El-Anwar MW, Elnashar I, Foad YA (2017) Platelet-rich plasma myringoplasty: a new office procedure for the repair of small tympanic membrane perforations. Ear Nose Throat J 96(8):312–326 28846786 10.1177/014556131709600818

[37] Nair NP, Alexander A, Abhishekh B, Hegde JS, Ganesan S, Saxena SK (2019) Safety and efficacy of autologous platelet-rich fibrin on graft uptake in myringoplasty: a randomized controlled trial. Int Archives Otorhinolaryngol 23(1):77–82 10.1055/s-0038-1649495 PMC6331294 30647788

[38] Haberman RS (2004) Middle ear and mastoid surgery. Thieme New York, NY, USA

[39] Sergi B, Galli J, De Corso E, Parrilla C, Paludetti G (2011) Overlay versus underlay myringoplasty: report of outcomes considering closure of perforation and hearing function. Acta Otorhinolaryngol Ital 31(6):366 22323847 PMC3272871

[40] Umapathy N, Dekker P (2003) Myringoplasty: is it worth performing in children? Archives Otolaryngology–Head Neck Surg 129(10):1053–1055 10.1001/archotol.129.10.1053 14568786

[41] Pfammatter A, Novoa E, Linder T (2013) Can myringoplasty close the air-bone gap? Otology Neurotology 34(4):705–710 23652328 10.1097/MAO.0b013e3182898550

[42] Wick CC, Arnaoutakis D, Kaul VF, Isaacson B (2017) Endoscopic lateral cartilage graft tympanoplasty. Otolaryngology–Head Neck Surg 157(4):683–689 10.1177/0194599817709436 28585463

[43] Gerlinger I, Ráth G, Szanyi I, Pytel J (2006) Myringoplasty for anterior and subtotal perforations using KTP-532 laser. Eur Archives Oto-Rhino-Laryngology Head Neck 263(9):816–819 10.1007/s00405-006-0077-z 16763822

[44] Vasani S, Whittaker M, Sharma P, Wong G, Patel S, Choa D (2010) Impact of modernising medical careers on operative training in otolaryngology. Clinical otolaryngology: official journal of ENT-UK; official journal of Netherlands Society for Oto-Rhino-Laryngology &. Cervico-Facial Surg 35(3):255–256 10.1111/j.1749-4486.2010.02144.x 20636765

[45] Kumar R (2013) A study of efficiency of autologous platelet rich plasma in myringoplasty. Kilpauk Medical College, Chennai

